# Experimental Study on the Fatigue Life of Low-Carbon Steel DD11: Combination of Pre-Strain and Notch Effect

**DOI:** 10.3390/ma19194136

**Published:** 2026-09-28

**Authors:** Ivan Tomasi, Luigi Solazzi, Candida Petrogalli, Alberto Mazzoni, Rodolfo Faglia, Giorgio Donzella

**Affiliations:** 1 Department of Mechanical and Industrial Engineering (DIMI), University of Brescia, Via Branze 38, 25123 Brescia, Italy; ivan.tomasi@unibs.it (I.T.); candida.petrogalli@unibs.it (C.P.); rodolfo.faglia@unibs.it (R.F.); giorgio.donzella@unibs.it (G.D.); 2 Moveero R&D Centre Europe, Via Molino Emili 18, Maclodio, 25030 Brescia, Italy; alberto.mazzoni@moveero.com

**Keywords:** experimental fatigue tests, rolled steel, numerical analysis, pre-strain condition, notch factor

## Abstract

**Highlights:**

- Pre-strain of 24% (beyond tensile strength) reduces the fatigue limit of DD11 steel by 8.5%.
- The combination of pre-strain and notch effect shows a negligible influence on the fatigue limit but an increase in the standard deviation.
- Double-notched geometry (K_t_ = 2.74) leads to localised plasticisation and a fatigue notch factor K_f_ = 1.39.

**Abstract:**

This paper is intended to study the fatigue behaviour of low-carbon steel DD11 with the combined effect of pre-strain and notch geometry, which are important in components working under cyclic loading in service. To this end, experimental fatigue tests were performed on specimens with different configurations: (i) specimens pre-strained to 24% (beyond the tensile strength point), (ii) specimens combining pre-strain and notch effects, and (iii) double-notched specimens, which feature a higher stress concentration factor. The experiments were complemented by numerical simulations and analytical formulations, and fracture surfaces were examined to support the interpretation of the results. The main results show that the applied pre-strain (24%) reduces the fatigue limit by 8.5% when compared to the un-notched case. However, when pre-strain and notch effects are combined, no significant additional influence on the fatigue limit is observed. In the double-notched configuration, a higher stress concentration factor is found compared to previous studies by the authors, leading to a reduction in the fatigue limit, with localised plasticisation similar to that observed in the compared scenario.

## 1. Introduction

Fatigue failure is a critical damage mechanism in engineering structures subjected to cyclic loading, where damage accumulates progressively and may lead to sudden fracture at stresses below the yield strength, observed in several mechanical components [1,2,3,4,5,6]. Driven by the propagation of flaws under cyclic loading, fatigue is inherently influenced by local stress concentrations [7]. Consequently, fatigue behaviour has been a central research topic for over a century—from Wöhler’s S-N characterisation to fracture mechanics-based frameworks.

Among the most widely used materials in mechanical engineering, low-carbon structural steels occupy a prominent role due to their favourable combination of strength, ductility, weldability, and cost-effectiveness. However, their fatigue response is sensitive to a number of factors that are frequently encountered in real service conditions, including geometric discontinuities [8], prior plastic deformation [9], and manufacturing processes such as cold forming [10]. Understanding how these factors individually and jointly modify the fatigue life of the material is therefore of both scientific and practical importance.

In particular, geometric discontinuities—such as holes, grooves, fillets, and cross-section changes—introduce localised stress amplification that substantially reduces fatigue resistance. Dantas et al. [11] found full notch sensitivity in S690 steel, with surface crack initiation dominating even in the very high cycle regime, where internal initiation is typically observed for smooth specimens. Moreover, a comparative study on S355 and S690 steels showed that while S690 has superior crack initiation resistance, its crack propagation resistance is lower; this inverse relationship implies that the advantage of high-strength steel may be lost in sharply notched details, which is where propagation dominates [12]. Notch severity and shape are decisive: Jagadesh et al. [13] reported fatigue life reductions up to 97% in V-notched specimens depending on notch geometry. The notch root stress gradient has been linked to this reduction, and recent models indicate that the characteristic length for critical distance approaches is a function of fatigue life, stress ratio, and notch geometry rather than a fixed material constant [14].

In addition to geometric discontinuities, prior plastic deformation—introduced during cold-forming, stamping, bending, or overloads—modifies the material’s microstructure through work hardening and generates non-uniform residual stresses, potentially altering the subsequent fatigue response [15]. The effect depends on the material, pre-strain level, and loading conditions. Reported trends are contrasting: Kusch et al. [16] observed cyclic hardening or softening depending on conditions; Taheri et al. [17] found a detrimental effect on fatigue life; Mroziński and Szala [18,19] reported a decrease in fatigue life and a new stabilisation state in S420M steel; conversely, some studies found an increase in fatigue strength under bending and torsion due to strain hardening [20]; others noted that low pre-strain produces negligible changes, while higher levels significantly reduce durability. For low-carbon ferritic-pearlitic steels—such as DD11—the dominant mechanisms are dislocation accumulation and substructure refinement, which increase cyclic yield stress but may reduce ductility and crack initiation resistance [21]. For TRIP and dual-phase steels, strain-induced martensitic transformation introduces additional strengthening with a critical pre-strain threshold governing the transition between work-hardening and transformation-dominated behaviour.

These two factors are fundamentally different in nature: geometric discontinuities act as stress raisers by modifying the local stress field, while prior plastic deformation alters the material’s microstructure through work hardening and, in some steels, phase transformations. Although both phenomena are well understood individually, their combined effect on fatigue behaviour has received considerably less systematic attention. This is particularly relevant in practical engineering scenarios, where the two factors frequently co-exist: pre-strained regions of cold-formed structural members often coincide with geometric stress concentrators such as bends, holes, or connection details. The interaction between residual stresses, microstructural changes induced by pre-straining, and the stress amplification produced by the notch geometry may result in a fatigue response that is not simply the superposition of the two individual effects.

To address this gap, this paper investigates the high-cycle fatigue limit of hot-rolled low-carbon structural steel DD11 (1.0332 according to UNI EN 10027-2:2015 [22]) under cyclic loading at R = 0.1. Three experimental configurations are systematically examined—all involving pre-straining and/or notch geometry—and compared with the reference condition of as-received smooth specimens, which were previously characterised by the authors [23]. Specifically, three configurations are investigated:

- Pre-strained smooth specimens (plastic deformation beyond ultimate tensile strength, ≃24% strain);
- Pre-strained specimens with a circular hole notch;
- Specimens with a more severe notch geometry exhibiting a higher stress concentration factor.

The novelty and contributions of this paper are threefold:

- It provides a systematic characterisation of the combined effect of large plastic pre-strain and notch severity on the fatigue limit of a low-carbon steel, quantifying how these factors interact rather than simply superimpose;
- It quantifies the scatter introduced by this combination, which is critical for reliability-based design and life assessment of components subjected to both manufacturing-induced pre-strain and geometric discontinuities;
- It offers practical design guidelines for industrial applications, such as agricultural and industrial wheels, where DD11 steel is commonly used for rim fabrication and where valve holes and other discontinuities are subjected to both plastic deformation and cyclic loading during service [24,25].

The findings also have broader relevance to other components—such as elastic cam systems [26]—where fatigue verification is essential despite constructive simplicity [27].

## 2. Materials and Sample Preparation

The specimen material is a low-carbon steel DD11 (1.0332 to the standard UNI EN 10027-2:2015 [22]). Table 1 shows the material’s chemical composition in compliance with UNI EN 10111:2008 [28]. The specimens were prepared such that their longest dimension was oriented perpendicular to the rolling direction. This indicates a behaviour that differs distinctly from that in the longitudinal direction, which is a consequence of the material’s manufacturing process. The location from which the samples were taken along the original strip was determined in accordance with ISO 377:2017 [29].

Each of the specimens used in this paper is directly machined from a hot-rolled DD11 steel sheet and has a rectangular cross-section and flat geometry.

In total, three categories of specimens were created: smooth, notched and double notched; Figure 1 depicts their geometries. This geometry was selected to fulfil the international standards for fatigue tests (ISO 12107:2012 [30]), the specifications of the testing apparatus and the geometry of the base material strip, which possesses a nominal thickness of 7 mm (as illustrated in section A-A of Figure 1). In particular, the specimens are depicted with the following nomenclature:

- The smooth specimen is defined as “PS”, having a gauge area of 4 × 7 mm;
- The notched specimen is defined as “PSN”, having a gauge area of 6 × 7 mm and a hole of 2 mm of diameter (the stress concentration factor resulted equal to 2.36, which is evaluated in the authors’ previous paper [23]);
- The double-notched specimen is defined as “DN”, having a gauge area of 8 × 7 mm, with a hole of 2.2 mm of diameter and a spherical countersink of 6 mm of diameter.

In particular, each specimen geometry was machined directly from the original strip—which has a thickness of 7 mm—using a computer numerical control (CNC) milling machine. Focusing on the “DN” specimen, to machine the specific gauge area, a 2.2 mm diameter hole was first drilled, and then a 6 mm diameter ball-end mill was used to create the countersink to a depth of 2 mm. The geometry differs from the standards only for the roughness of the surfaces: since one of the goals of this paper is to analyse the material as it is implemented in the mechanical component, some of the surfaces of the specimen were not machined, reaching a higher level of roughness than the one defined in the standards. The roughness at the non-machined surfaces was evaluated through the roughness tester Mitutoyo SJ-201, evaluating a roughness of 7.72 μm and a deviation standard of 1.28 μm.

To understand the results of this paper, Table 2 defines the values obtained by the previous experimental study [23].

## 3. Experimental Analysis

### 3.1. Test Machines for Uniaxial Fatigue Tests

Fatigue tests were performed using a Rumul Mikrotron resonant testing machine (manufacturer: Russenberger Prüfmaschinen AG, Neuhausen am Rheinfall, Switzerland) with a maximum load capacity of 20 kN, as illustrated in Figure 2. To apply the pre-strain condition to the specimens, an Instron 8501 servo-hydraulic universal testing system (Instron, Norwood, MA, USA; supplied by Instron–ITW Test & Measurement Italia S.r.l., Pianezza, Italy) was adopted. This test machine has a maximum load capacity of 100 kN, and pre-strain was applied using displacement control. The tests were conducted at the Fatigue Testing Laboratory of the University of Brescia, Italy.

### 3.2. Fatigue Tests Performed

Axial fatigue tests with a sinusoidal load function and a load ratio of R = 0.1 were conducted at a frequency of about 80 Hz. The frequency represents the number of cycles per second, which is determined automatically by the specimen stiffness and the testing machine parameters, including the masses, the internal springs of the machine, and the electromagnet that imposes the oscillatory motion. The requirement to prevent compression and restriction on the maximum stress to preserve the same failure mode, respectively, were the driving forces behind these specific load ratio values. The staircase approach is used for every situation that is examined because the primary objective of this paper is to determine the material’s fatigue limit under various conditions.

The specimen’s total fracture was taken to be the failure criterion. However, the tests were halted and deemed runouts if specimens reached 2,000,000 cycles. The choice of this specific number is justified by its widespread adoption in the literature (e.g., [31,32,33]). This paper examined these different conditions:

- The fatigue limit of the material with a 24% pre-strain level was determined using the staircase method. This value was chosen to investigate the fatigue behaviour of the material beyond its ultimate tensile strength. Typically, the static behaviour of materials with such high pre-strain levels has been the primary focus of study [19]. This pre-strain level was achieved by stretching the specimen to a displacement of 7 mm and then unloading it;
- The fatigue limit of the material, considering the combination of pre-strain and notch effect, is determined using the staircase method. The level of localised plasticisation is defined as 31 %, and it is evaluated through the finite element method as observed in Section 5. This pre-strain level was achieved by stretching the specimen to a displacement of 0.5 mm and then unloading it;
- The fatigue limit of the material, considering the presence of the notch effect with a stress concentration factor higher than the one of authors’ previous research study [23].

To understand the staircase method, the example of the pre-strained condition is here reported in Table 3 and Table 4. A staircase method is usually defined by a minimum of 15 specimens, as shown in the example, following the standard for a fatigue test [30].

The staircase technique tests, their amplitude stress, and whether the test was a runout (designated with “O”) or the specimen attained failure (designated with “X”) are displayed in Table 3. Because each test in this approach depends on the one before it, if the prior specimen failed, the subsequent test should be conducted at a lower level of stress amplitude; conversely, if the prior specimen was a runout, the subsequent test should be conducted at an upper level of stress amplitude. The results are explained in Table 4, where the level begins at 0, the lowest level of stress amplitude (in this case, 125 MPa), and the values to evaluate the coefficient *C*, *A*, and *B* are taken based on the least number of events $f_{i}$. In this case, the runout tests (“O”) are chosen because they are less than the failure ones. The sum of the selected events is represented by coefficient *C*, the sum of each event multiplied by its level of stress amplitude is represented by coefficient *A*, and the sum of each event multiplied by the square of its level of stress amplitude is represented by coefficient *B*. Equation (1) [30] requires these coefficients in order to calculate the fatigue limit $\sigma_{a50}$ and its standard deviation (Std. Dev.).

(1) $$\sigma_{a50}=\sigma_{0}+\Delta S\left(\frac{A}{C}\pm 0.5\right);\;\;\;\;\mathrm{Std}.\mathrm{Dev}.=1.62\cdot \Delta S\left(\frac{C\cdot B-A^{2}}{C^{2}}+0.029\right)$$

Here, $\sigma_{0}$ denotes the lowest stress amplitude level of the staircase method, ΔS the stress amplitude step (here taken as 2.5 MPa), and the sign before 0.5 in Equation (1) depends on the event considered: it is negative for tests that reached failure and positive for runouts. The alphanumeric coding used to identify the specimens in this paper is presented in Table 5. Specimens labelled “PS” were adopted to investigate the fatigue limit of the material with pre-deformation. To investigate the combination of pre-strain and notch effect, specimens with code “PSN” were used, while for the specimens with a different notch, the code “DN” labels them.

It is essential to emphasise that positive load ratios are selected to prevent potential buckling, which may occur with a negative load ratio due to compression effects. Additionally, fractography analysis is presented to comprehensively elucidate the material’s behaviour under fatigue loads.

## 4. Numerical Simulations

With the aim of generalising the experimental results, numerical analyses were carried out using the finite element method (FEM). These simulations were performed with Ansys® 2025 R1, and for the smooth specimen, a solid mesh of around 80,000 quadratic hexahedral elements (HEX20) and 350,000 nodes is implemented, as shown in Figure 3a. For the notched specimen, a solid mesh consisting of around 360,000 quadratic hexahedral (HEX20) and tetrahedral (TET10) elements and 1,500,000 nodes is implemented, as shown in Figure 3b. For the double-notched specimen (DN specimen), a solid mesh of around 140,000 quadratic tetrahedral elements (TET10) and 237,000 nodes is defined, as represented in Figure 3c. In particular, a mesh refinement is executed in the specimen gauge section, where the average dimension of the element is putted to 0.1 mm, while for the grip section, an element average size is defined equal to 0.75 mm, since it is not the interest of the authors to analyse this area.

In the numerical simulations performed, the main goal was to understand the mechanical behaviour of the specimens during the first load cycle. Therefore, the pre-strain phase was numerically investigated for the specimens labelled “PS” and “PSN”, while a numerical study on the stress concentration factor was carried out for the “DN” specimen.” Regarding the boundary conditions, Figure 4 illustrates where the fixed constraints and the controlled forces/displacements are applied in order to depict the real behaviour of the specimen stressed on the experimental machine. Because of this arrangement, the mesh’s refinement at the boundary of the fixed constraint can be justified; nevertheless, since the gauge area is the one being examined, that portion of the specimen was not relevant to this paper. There were three boundary conditions for the numerical simulations:

- For the smooth specimen, the displacement imposed was 7 mm, and when this displacement is reached, the specimen was unloaded;
- For the notched specimen, the displacement imposed was 0.5 mm, and when this displacement is reached, the specimen was unloaded;
- For the DN specimen, since the aim is to analyse the elastic behaviour of the material, a force equal to 3000 N was applied.

In particular, regarding the “PS” and the “PSN” specimens, the displacement control was imposed through a curve that represents the experimental setup executed.

## 5. Results and Discussion

This section analyses the results obtained by the numerical analyses and experimental tests. For a better comprehension of the results, Figure 5 shows the curve σ−ε of the material with the pre-strain implemented for the “PS” specimens.

### 5.1. Results of the Numerical Simulations

Starting with the numerical analysis of the pre-strain on the smooth specimen, Figure 6 shows the equivalent plastic strain and the normal stress at the end of the phase of pre-strain. As it can be seen, all of the gauge lengths reached a high level of plasticisation of the material with a value around 24%. To be specific, in accordance with the curve σ−ε of the material shown in Figure 5, all the area is beyond the ultimate tensile strength point, meaning that the specimen is at the beginning of the necking phase. Regarding the stress along the axis of application of the boundary condition, it is possible to notice a positive stress up to 90 MPa on the surface of the specimen, while on the edges, the stress observed is around 10 MPa. These residual stresses are redistributed across the cross-section upon unloading the specimen, explaining the coexistence of tensile and compressive zones.

From this numerical simulation, it could be assumed that crack initiation could start in the middle of the faces of the gauge length. However, as also shown in Section 5.3, the fatigue crack phenomena started at the edges of the gauge length. The reason behind this difference could be due to several main factors. One of them is the fact that the roughness of the specimen is a peculiar contributor to crack initiation. Specifically, the roughness was found to be 7.72 μm, which is higher than the standard limit of 0.8 μm, but since the goal of this paper is to analyse the material as it is implemented in the mechanical component, the surface could not be machined. Another factor that could influence the material’s properties is the effect of the production process of the steel sheets from which the specimens were obtained.

About the notched specimen, Figure 7 shows the equivalent plastic strain and the normal stress at the end of the phase of pre-strain. The stress concentration factor for this geometry is equal to 2.36, as evaluated in the authors’ previous paper [23]. Focusing on the plastic strain, a localised plasticisation of 31% can be seen on the surface of the hole, while as for the residual normal stresses, there are peaks of compression at the edge of the hole, but tensile stresses on the gauge length on the top and the bottom, up to 145 MPa. However, that area during the fatigue tests is less stressed due to the increase in the area. This simulation also predicts the beginning of the crack: the fracture of the specimen in the experimental tests was not seen in the exact middle of the specimen but rather usually from the edge of the hole where the residual normal stress changes from negative to positive values. In this specific location, the stress varies from −10 MPa to 5 MPa.

The stress applied to the DN specimen FEM simulation was through a tensional force equal to 3000 N to remain in the elastic field of the material. On this specimen, the stress evaluated analytically without considering the stress concentration factor (over a gauge area of 32.6 ${\mathrm{mm}}^{2}$) was 92 MPa. This represents the nominal stress, defined as the average stress acting on the net cross-section at the hole position, while for the notched specimen, the value numerically evaluated is equal to 252 MPa, as can be seen from the FEM results shown in Figure 8. To evaluate the stress concentration factor $K_{t}$ [34], Equation (2) is here presented:

(2) $$K_{t}=\frac{\sigma_{\max,n}}{\sigma_{n}}$$

Here, $\sigma_{\max,n}$ is the stress value at the edge of the hole, while $\sigma_{n}$ is the nominal stress calculated at a location without a hole. The resulting value of $K_{t}$ was 2.74.

### 5.2. Results of the Fatigue Tests

In order to examine the impact of pre-strain on the fatigue limit, a group of smooth specimens were subjected to a pre-strain level of tensile plastic deformation equal to 24%. The pre-strain level imposed on the smooth specimen is depicted in Figure 5, which reports the experimentally derived σ−ε curve of the material together with the strain position. The specimens in this group are labelled “PS”. Because this level of pre-strain is located on the plateau of the σ−ε curve, the stretching process was conducted using displacement control.

Regarding the notched tests, the fatigue limit was analysed. In particular, the specimens with denomination “PSN” were tested and compared to the specimens without the pre-strain condition. Considering that the level of the pre-strain measured is very close to the static fracture of the material, the pre-strain phase was defined using the displacement control also in this scenario.

A comparison of all the fatigue limits examined and the fatigue limit of the specimen without the pre-strain condition is presented in Table 6. A stress ratio of R=0.1 was used for all tests; however, distinct stress steps (ΔS) were used for the staircase approach. According to the standards [30], a step of roughly 5% of the projected mean fatigue strength may be utilised as the stress step if the standard deviation is unknown. Therefore, the material’s stress step should be between 5 and 10 MPa. Experimentally, it was observed that in the scenario of the pre-strained notched specimens (PSNs), the variables implied (deformation imposed and mechanical processing) could not allow a stress step of 5%, but a little higher, around 8%, imposing a stress step of 10 MPa. For the “R1” specimens, which are the base material with stress ratio R = 0.1, the step was even lower than 5% due to the missing of all these variables; the same reasoning can be applied to specimen “N”, which are notched specimen already analysed previously and having the same geometry of PSN specimens. The table shows also the coefficient of variation (C.O.V.), which can be evaluated through Equation (3) [35,36]:

(3) $$C.O.V.\%=\frac{\mathrm{Std}.\mathrm{Dev}.}{\sigma_{\mathrm{fa}}}\cdot 100$$

where $\sigma_{\mathrm{fa}}$ and Std.Dev. are the fatigue limit of the material at the observed condition and its standard deviation, respectively. This variable measures the dispersion of data points around the mean value, and it is a dimensionless number.

The values indicate that the pre-deformation of the specimen presents a decrease in the fatigue limit strength, which is probably due to the initial necking of the material. To understand this reduction, Equation (4) shows the percentage of reduction in the fatigue limit:

(4) $$Red_{\sigma_{a}}\%=\frac{\sigma_{\mathrm{fa},R1}-\sigma_{\mathrm{fa},\mathrm{PS}}}{\sigma_{\mathrm{fa},R1}}\cdot 100$$

where $\sigma_{\mathrm{fa},R1}$ is the fatigue limit of the material without the pre-strain condition at a level of stress ratio *R* equal to 0.1, while $\sigma_{\mathrm{fa},\mathrm{PS}}$ is the fatigue limit of the material with a level of pre-strain equal to 24% and with the same level of stress ratio *R*. A fatigue limit reduction of 8.5% is also observed. A reduction in the fatigue life of the material brought by the pre-strain effect has been noted in a previous study [37], where the fatigue life of X80 steel was investigated under different levels of pre-strain. As for the C.O.V., it can be noted that the value increases when the pre-strain condition is applied due to the presence of the new variable.

Regarding the combined effect of pre-strain and notch on the fatigue behaviour of the material, it can be observed that the pre-strain condition has a negligible influence on the notch fatigue limit strength. Indeed, the fatigue limit of the PSN specimen (121.67 MPa) is very close to that of the N specimen (121.25 MPa), suggesting that the pre-strain does not significantly alter the material’s resistance to fatigue under the tested conditions. However, the standard deviation for the PSN specimens is considerably higher (9.47 MPa) compared with the other configurations, indicating greater scatter in the experimental results. This increase in variability can be attributed to the combined presence of both pre-strain and notch, which introduces additional sources of uncertainty. As supported by the numerical simulations, the crack initiation site in the PSN specimen is located in a region characterised by residual stress gradients, specifically within a range between −10 and +5 MPa, as previously discussed. This local stress heterogeneity, arising from the interaction between the pre-straining operation and the notch geometry, reasonably explains both the increased standard deviation and the unchanged fatigue limit. Furthermore, the coefficient of variation (C.O.V.) for the PSN specimen reaches 7.78%, the highest value among all tested groups, which further confirms that the simultaneous presence of pre-strain and notch amplifies the dispersion of the experimental data. A similar result was reported in a previous research paper [38], where the fatigue life of dual-phase steel DP600 (designated 1.0941 according to UNI EN 10027-2:2015 [22]) was tested, observing the notch factor and pre-strain effects simultaneously. In the cited study, after pre-straining, the notch fatigue results showed a negligible improvement in fatigue life.

The fatigue strength concentration factor was examined by contrasting the material’s fatigue limit with the DN specimens’ fatigue limit. The fatigue notch factor $K_{f}$ can be evaluated using Equation (5) [39]:

(5) $$K_{f}=\frac{\sigma_{\mathrm{fa},\mathrm{smooth}}}{\sigma_{\mathrm{fa},\mathrm{notched}}}$$

Here, $\sigma_{\mathrm{fa},\mathrm{smooth}}$ is the unnotched fatigue limit of the material, while $\sigma_{\mathrm{fa},\mathrm{notched}}$ is the fatigue limit of the material accounting for the notch effect. The resulting value of $K_{f}$ was 1.39.

In order to comprehend this result, a material parameter denoted as α in Equation (6) needs be used to estimate the value of the fatigue notch sensitivity factor *q*:

(6) $$\log\alpha =-1.079\cdot 10^{-9}R_{m}^{3}+2.740\cdot 10^{-6}R_{m}^{2}-3.740\cdot 10^{-3}R_{m}+0.6404$$

Here, $R_{m}$ is the tensile strength of the material, and the value of α was found to be 0.632. Using this value, it is now possible to evaluate the fatigue notch sensitivity factor *q* by means of Equation (7):

(7) $$q=\frac{1}{1+\sqrt{\frac{\alpha }{\rho }}}$$

In this case, ρ is the notch radius, which is 1.1 mm in the geometry under consideration, and α was previously calculated. This indicates that the notch sensitivity factor *q* is 0.569. Equation (8) can be used to determine the expected value of the elastic fatigue notch factor $K_{f,\mathrm{el}}$ (the fatigue notch factor of the studied scenario if the stress cycle is under the yield strength of the material), given that the value of $K_{t}$ as previously described is equal to 2.74.

(8) $$K_{f,\mathrm{el}}=1+q\cdot \left(K_{t}-1\right)=1.99$$

Compared to the $K_{f}$ discovered experimentally, this finding is significantly higher. The material used and the stress conditions of the tests conducted could be the causes of the fatigue notch factor’s decline. As previously shown in the literature [40,41,42], there are several reasons why $K_{f}$ may decrease:

- The material enters the elasto-plastic field due to the loads applied and the chosen stress ratio (R =0.1);
- Because of the material’s high ductility and the shape used, notch blunting phenomena can easily occur;
- When operating in the high-cycle and very-high-cycle fatigue regimes, repeated plastic strain can accumulate, which prevents cracks from propagating.

In a previous research study [43], it was found that the mean stress effect on fatigue life is significantly reduced in notched specimens of low-alloy steel due to local mean stress relaxation at the notch root, which is caused by cyclic plastic deformation. This relaxation occurred because the material underwent localised plastic straining that progressively reduced the local mean stress towards zero, making the applied mean stress less influential on fatigue life. In fact, using Equation (9), one can determine the maximum stress during the fatigue limit cycle $\sigma_{\max,\lim}$, given that $\sigma_{a,\lim}$ was found to be 121.25 MPa for the notched specimen and the stress ratio R was 0.1:

(9) $$\sigma_{f,\max}=\frac{2\cdot \sigma_{\mathrm{fa}}}{1-R}=227.78\;\;\mathrm{MPa}$$

Here, *R* is the stress ratio. Thus, the maximum local stress can be evaluated by means of Equation (10), using the stress concentration factor $K_{t}$:

(10) $$\sigma_{\max,\mathrm{loc}}=K_{t}\cdot \sigma_{f,\max}=624.12\;\;\mathrm{MPa}>\sigma_{y}=287.56\;\;\mathrm{MPa}$$

This means that yielding occurs around the notch area, because the maximum local stress $\sigma_{\max,\mathrm{loc}}$ is greater than the yield strength of the material $\sigma_{y}$. To be more specific, since the so-called reversed yielding condition is not satisfied, this condition is called initial yielding [44]. Under these circumstances, the fatigue notch factor for mean stress $k_{\mathrm{fm}}$ can be defined using Equation (11):

(11) $$k_{\mathrm{fm}}=\frac{\sigma_{y}-K_{t}\cdot \sigma_{\mathrm{fa}}}{|\sigma_{\mathrm{fm}}|}=0.054$$

Here, $\sigma_{\mathrm{fa}}$ and $\sigma_{\mathrm{fm}}$ are the alternating and mean stress of the notched fatigue limit cycle, respectively. So, a nearly reversed yielding condition is described by the result (in which the fatigue notch factor for mean stress $k_{\mathrm{fm}}$ equals 0), leading to the following stresses at the notch area:

(12) $$\sigma_{a,\mathrm{loc}}=K_{t}\cdot \sigma_{\mathrm{fa}}=280.85\;\;\mathrm{MPa}\;;\;\;\;\;\sigma_{m,\mathrm{loc}}=k_{\mathrm{fm}}\cdot \sigma_{\mathrm{fm}}=6.77\;\;\mathrm{MPa}$$

Here, $\sigma_{a,\mathrm{loc}}$ and $\sigma_{m,\mathrm{loc}}$ are, respectively, the alternate and the mean stress of the notch fatigue limit cycle localised in the plasticised area.

This mechanical behaviour is quite similar to that demonstrated in the authors’ previous paper with a different value of stress concentration factor, confirming the mechanical behaviour with another level of $K_{t}$.

### 5.3. Macroscopic Observations of the Fatigue Fracture Surfaces

This section investigates the fracture surface of the specimen in the different conditions analysed in terms of the damaged area and the final rapid-fracture area. At the end of each test, the specimens’ fracture surface was acquired with a Nikon SMZ1000 stereomicroscope equipped with an Alexasoft Digital camera and image analyser software. Figure 9 and Figure 10 show an example of one of the fracture surfaces analysed for each condition, and the most relevant fracture regions have been marked and identified wherever they could be unambiguously distinguished. In particular, Figure 9 shows one of the fracture surfaces analysed for the smooth specimens in two conditions: with and without pre-strain. Figure 10 shows the fatigue fracture surfaces of notched specimens. The main goal of these figures is to understand the correlation between the different area of the gauge section failed through static and fatigue phenomena.

To evaluate the percentage of surface failed by fatigue, Inkscape 1.4.2 software was adopted for the image analyses. In these evaluations, the average percentage of the fracture surface and its standard deviation were computed. The specimens were chosen based on their stress amplitude, which was close to the fatigue limit evaluated. Table 7 presents a summary of this analysis.

For a better comprehension, Figure 11 shows a graph correlating the stress amplitude applied to the specimen and the percentage of area failed by fatigue. It is clear that the percentage of area failed by the effect of fatigue decreases when the stress amplitude increases, and there is a linear correlation between the two parameters.

## 6. Conclusions

The combined effect of pre-strain and notch geometry on the fatigue behaviour of DD11 low-carbon steel flat bars was investigated in this paper. The experimental campaign, supported by numerical simulations, has resulted in the following main findings:

- The level of pre-strain of 24%, which is above the tensile strength of the material, results in a reduction in the fatigue limit of about 8.5% in comparison with the untreated condition;
- The pre-strain effect on the fatigue limit is negligible for the case with a notch. However, the simultaneous occurrence of both phenomena adds additional sources of variability, as indicated by the increase in the standard deviation of the fatigue data;
- A similar mechanical behaviour was observed for a different notch geometry characterised by a stress concentration factor $K_{t}$ = 2.74, confirming the low fatigue sensitivity of this material under notched conditions. This behaviour may be attributed to a number of factors, including the ductility of the material, the method of fabrication of the specimen, and the test conditions, which promote localised plasticisation at the notch root.

The results obtained are useful for the design of mechanical components, particularly for cold forming processes and in the presence of geometric discontinuities. The variability of the mechanical response for the combined pre-strain and notch case is an essential component of ductile materials, such as DD11 steel, which has to be considered carefully in design and quality control. Future work will examine methods to reduce the notch effect on fatigue performance. Possible approaches include the application of a stamping process to induce local plasticisation around the hole or the introduction of auxiliary smaller holes aligned with the loading direction. Additionally, the same conditions examined in this paper under uniaxial loading will be extended to bending fatigue configurations. Finally, the combined effect of pre-strain and notch will be investigated on less ductile materials to assess the generalisability of this paper’s results.

## Figures and Tables

**Figure 1:**
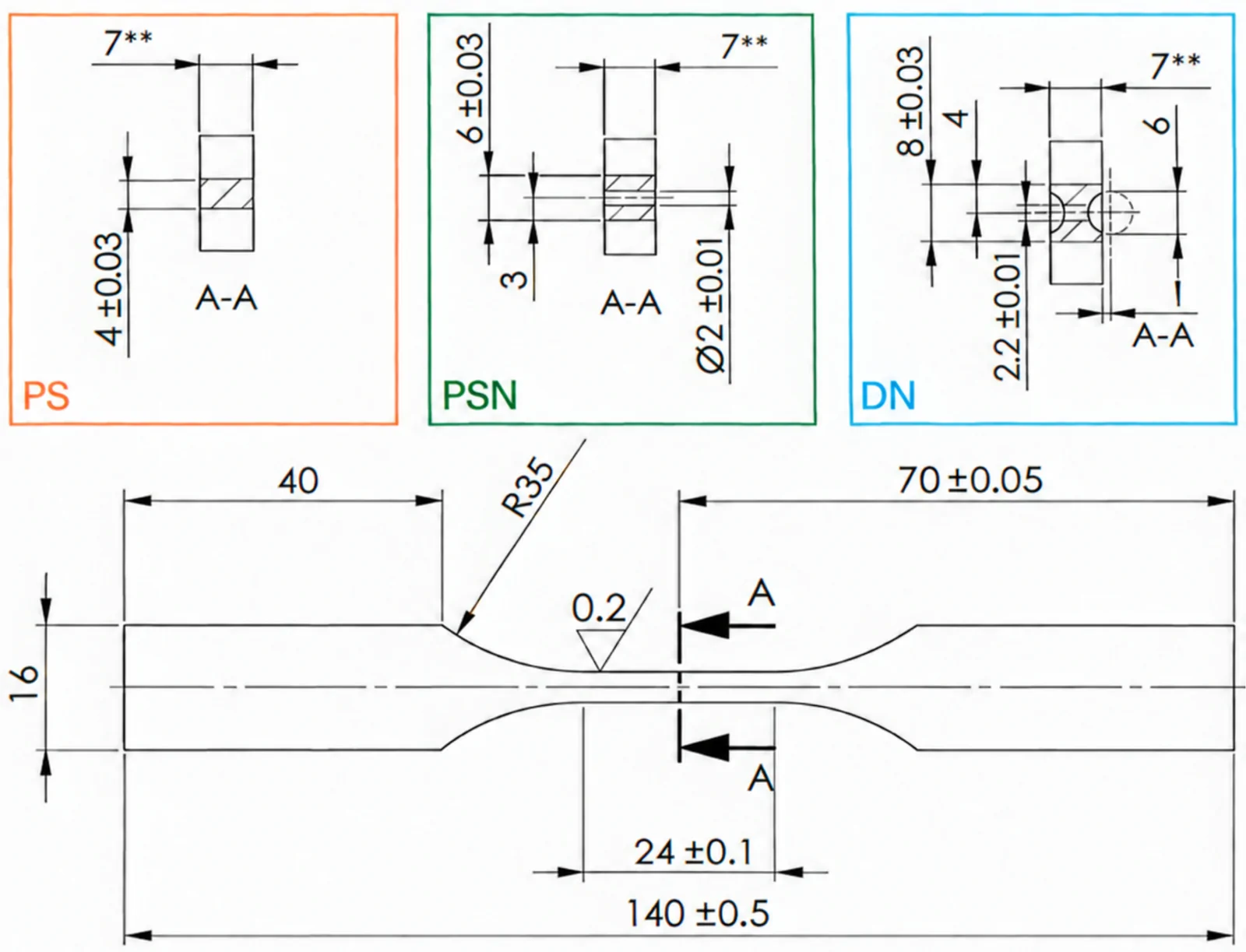
Technical draw of the specimens adopted for the fatigue tests: the section differs for each type of specimen. (**) 7 mm is the specimen’s thickness, which comes directly from the metal sheet obtained through the production process.

**Figure 2:**
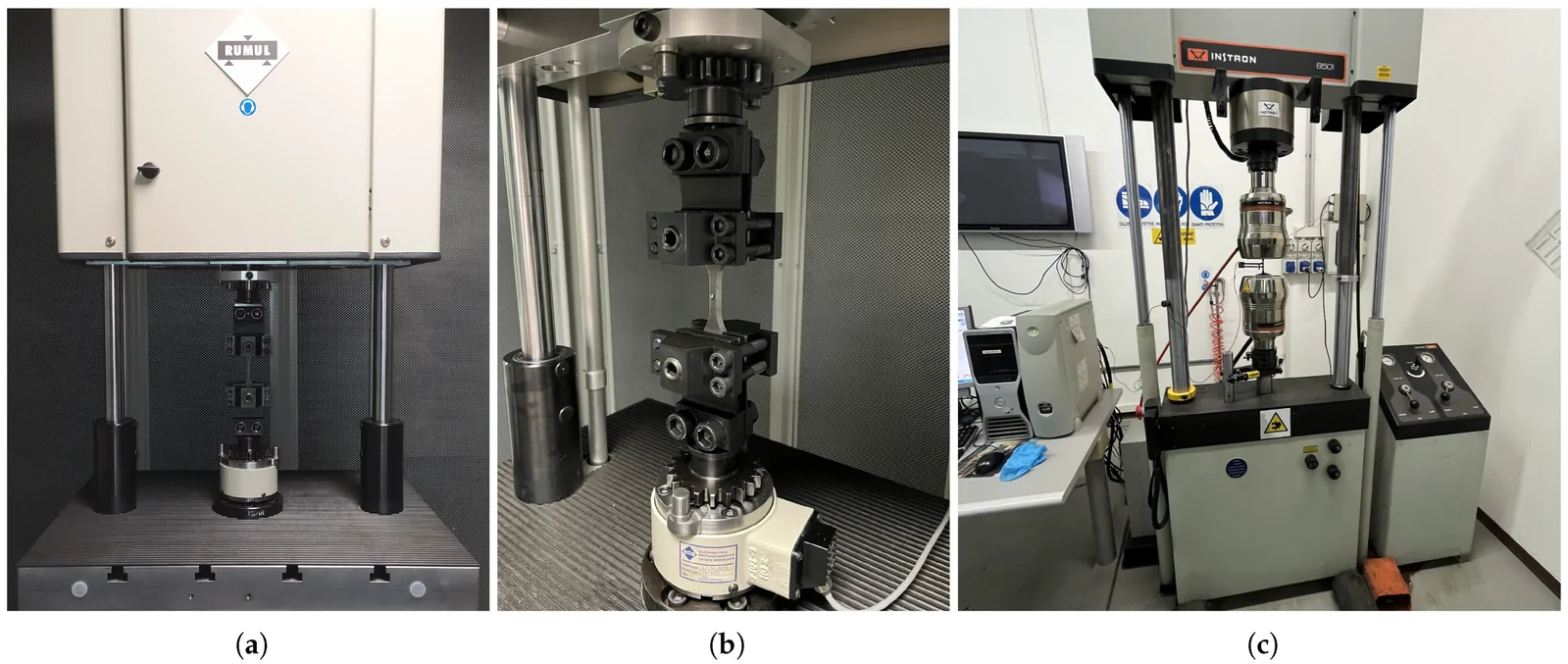
Photos of the experimental test machine. (**a**) Resonant test machine. (**b**) Fatigue test setup. (**c**) Instron test machine.

**Figure 3:**
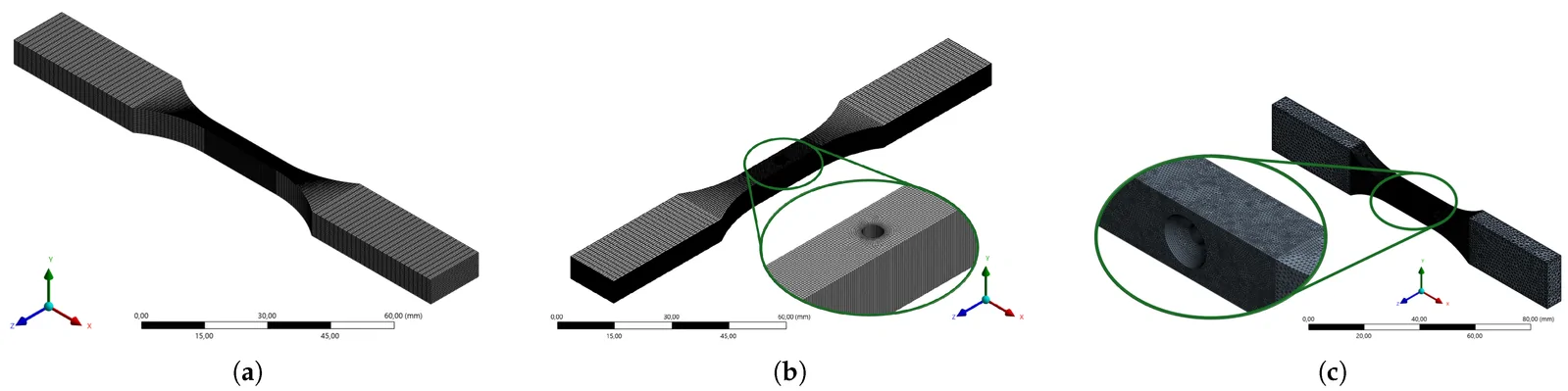
Meshes adopted. (**a**) Mesh of the smooth specimen. (**b**) Mesh of the notched specimen. (**c**) Mesh of the double-notched specimen.

**Figure 4:**
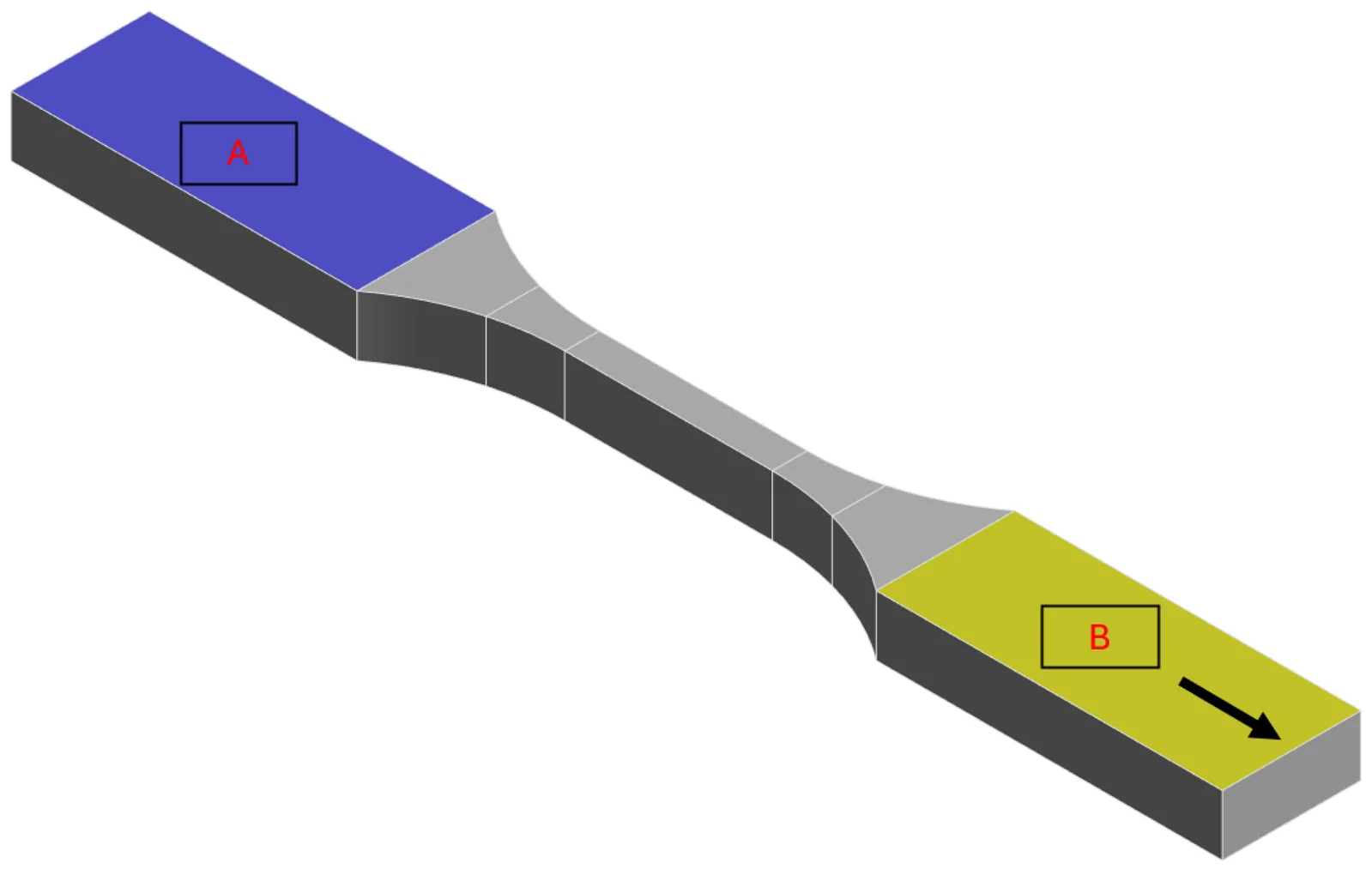
Boundary conditions of the specimen analysed: the letter A defines the fixed supports, while B defines the displacements/forces applied. The surfaces of application are also on the opposite face, in order to have a symmetrical effect on the specimen, in accordance with the experimental setup.

**Figure 5:**
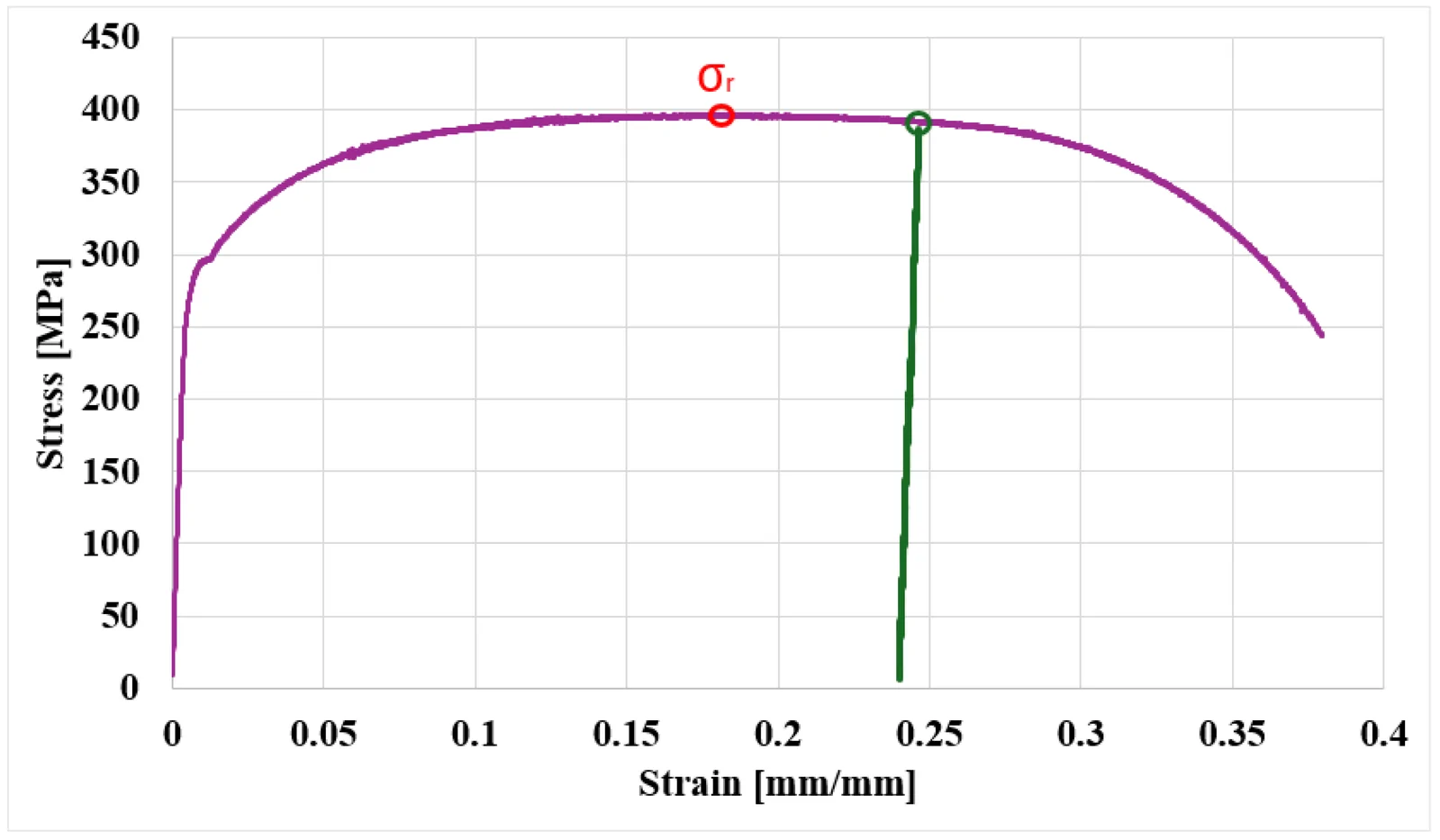
Curve σ−ε of the material studied with the position of the strain applied to the specimen PS.

**Figure 6:**
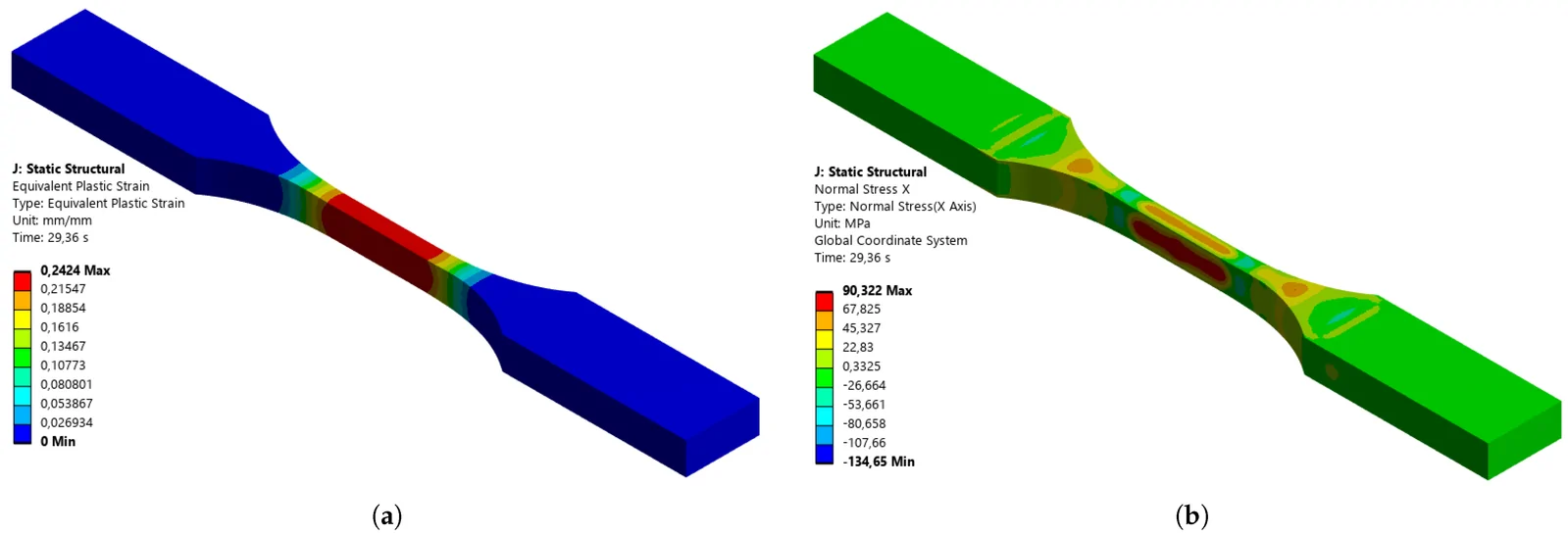
Numerical results on the pre-strain phase on smooth specimen. (**a**) Equivalent plastic strain. (**b**) Normal stress along the axis of the applied displacement.

**Figure 7:**
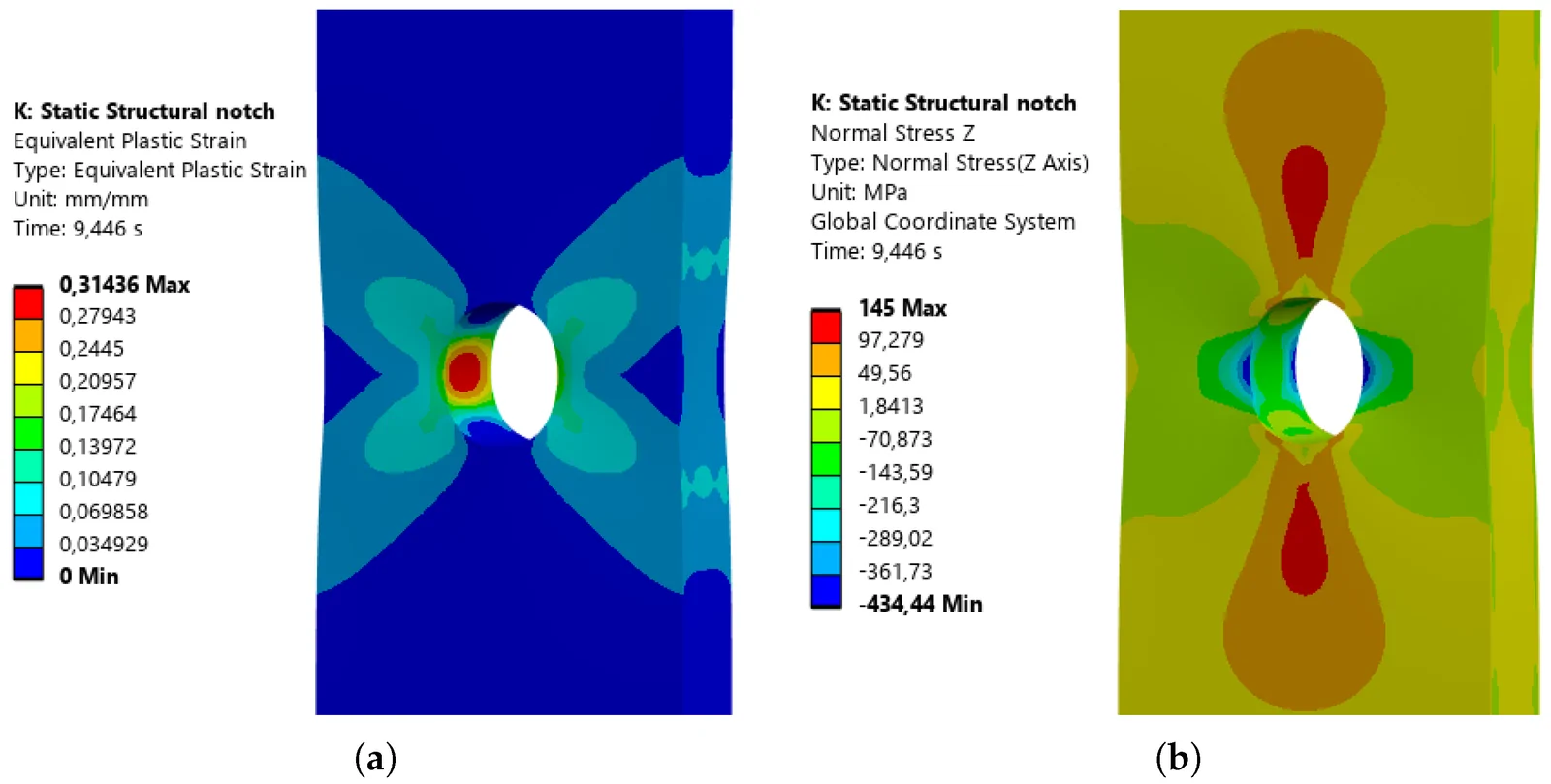
Numerical results on the pre-strain phase on notched specimen. (**a**) Equivalent plastic strain. (**b**) Normal stress along the axis of the applied displacement.

**Figure 8:**
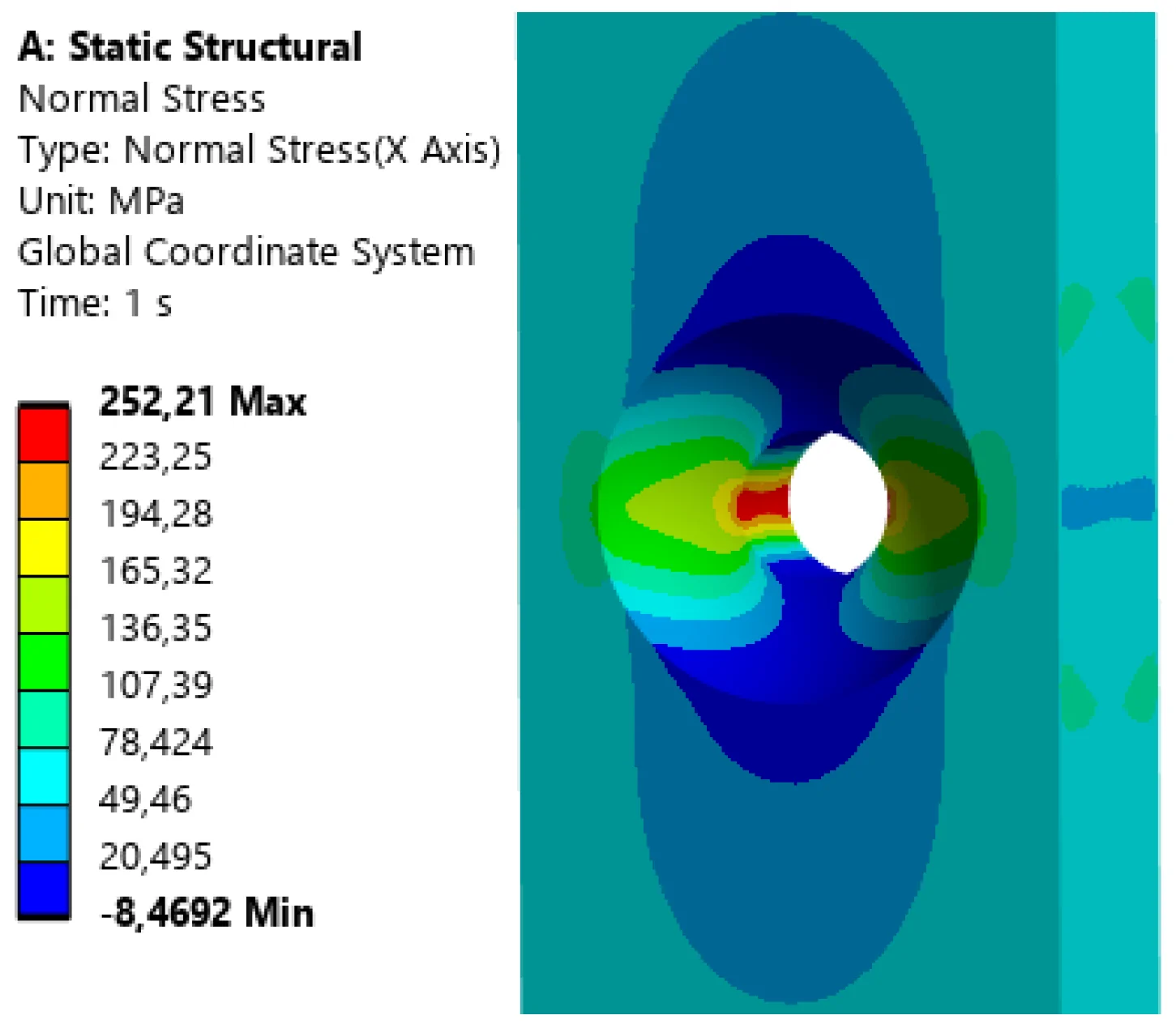
Numerical results of the linear static simulation on the double-notch specimen (DN): normal stress along the axis of the applied force.

**Figure 9:**
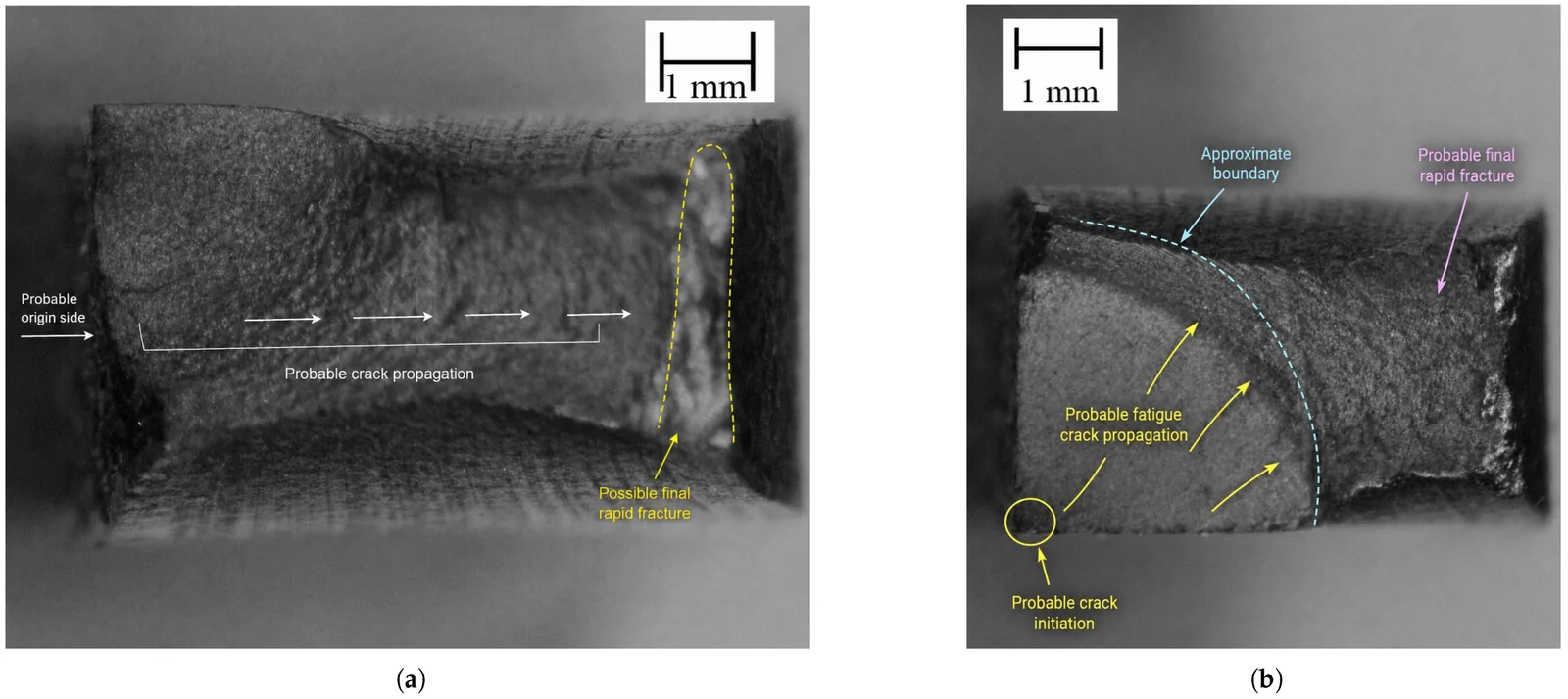
Fracture images of the analysed conditions for smooth specimens. (**a**) Fracture image of the R1 specimen. (**b**) Fracture image of the PS specimen.

**Figure 10:**
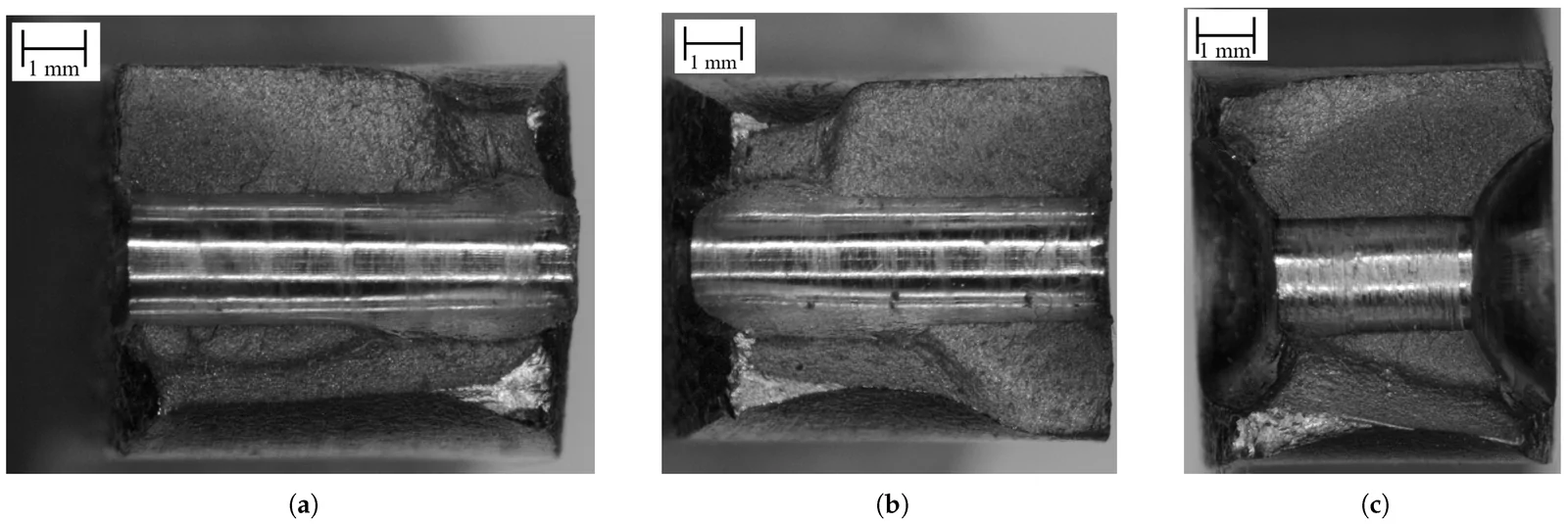
Fracture images of the analysed conditions with notch factor. (**a**) Fracture image of the N specimen. (**b**) Fracture image of the PSN specimen. (**c**) Fracture image of the DN specimen.

**Figure 11:**
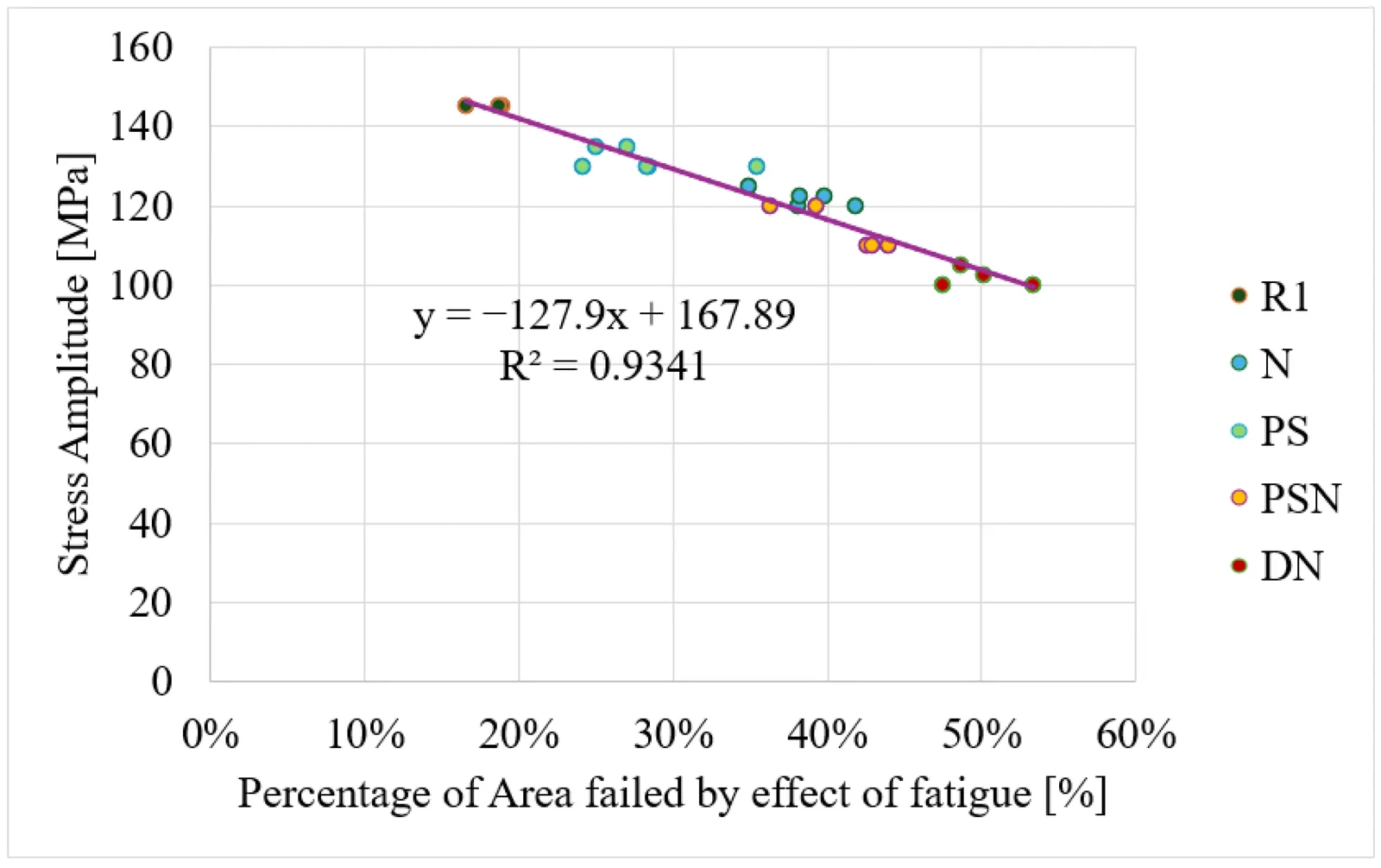
Stress amplitude—percentage of area failed by the effect of fatigue chart.

**Table 1:** Chemical composition of the DD11 (wt%).

C	Mn	P	S	Si	Al	Cu	Ni	Cr	N
0.07	0.42	0.010	0.008	0.05	0.016	0.030	0.012	0.020	0.004

**Table 2:** Mechanical properties of the material experimentally evaluated.

Mechanical Property [MPa]	Symbol	Value
Young’s modulus	E	216,000
Tensile strength	$\sigma_{r}$	395
Yield strength	$\sigma_{y}$	287
Fatigue limit with R = 0.1	$\sigma_{\mathrm{fa},\mathrm{bm},\;R\;=\;0.1}$	143
Fatigue limit of notched specimen with R = 0.1	$\sigma_{\mathrm{fa},n,\;R\;=\;0.1}$	121
Fatigue limit of pre-strain specimen (11%) with R = 0.1	$\sigma_{\mathrm{fa},\mathrm{ps}11\%,\;R\;=\;0.1}$	144

**Table 3:** Staircase test procedure of the pre-strained PS specimens.

$\sigma_{a}$	Specimen ID														
[MPa]	PS1	PS2	PS3	PS4	PS5	PS6	PS7	PS8	PS9	PS10	PS11	PS12	PS13	PS14	PS15
140							X								
135						O		X				X		X	
130	X		X		O				X		O		O		X
125		O		O						O					
$N_{f}/10^{6}$	1.52	2.0	1.07	2.0	2.0	2.0	0.49	1.63	1.13	2.0	2.0	1.03	2.0	1.19	1.50

The marker “X” denotes a specimen that reached failure, while the marker “O” indicates a runout specimen.

**Table 4:** Evaluation of the fatigue limit of the notch PS specimens.

Stress [MPa]	Level	Events		Values		
$S_{i}$	i	X	O	$f_{i}$	${\mathrm{if}}_{i}$	$i^{2}f_{i}$
140	3	1	0	0	0	0
135	2	3	1	1	2	4
130	1	4	3	3	3	3
125	0	0	3	3	0	0
Sum	-	8	7	C=7	A=5	B=7

**Table 5:** Legend of the letters used for specimen identification.

ID	#	Purpose
PS	15	Fatigue tests on axially deformed specimens ($\epsilon_{p}=24\%$)
PSN	15	Fatigue tests on notched axially deformed specimens ($\epsilon_{p},loc=31\%$)
DN	15	Fatigue tests on notched specimens

The column # defines the number of specimens tested for each configuration.

**Table 6:** Summary of the fatigue limits results.

Specimen ID	$\sigma_{\mathrm{fa}}$ [MPa]	Std. Dev. [MPa]	C.O.V [%]	ΔS [MPa]
R1 ^1^	143.25	0.77	0.54	2.5
N ^1^	121.25	2.43	2.01	2.5
PS	131.07	4.21	3.46	5
PSN	121.67	9.47	7.78	10
DN	102.5	4.86	4.74	5

^1^ These values are taken from the authors’ previous paper.

**Table 7:** Summary of the fracture surface analysis. $A_{f,\mathrm{avg}}\%$ is the percentage of area failed by the effect of fatigue.

Specimen ID	$A_{f,\mathrm{avg}}$ [${\mathrm{mm}}^{2}$]	$A_{\mathrm{tot}}$ [${\mathrm{mm}}^{2}$]	$A_{f,\mathrm{avg}}\%$ [%]	Std. Dev. [%]
R1 ^1^	5.06	28	18.1	1.3
N ^1^	10.81	28	38.6	2.5
PS	7.86	28	28.1	4.0
PSN	11.48	28	41.0	3.2
DN	16.27	32.6	49.9	2.6

^1^ These values are evaluated from the specimens observed in the authors’ previous paper [23].

## Data Availability

The original contributions presented in this study are included in the article. Further inquiries can be directed to the corresponding author.

## Abbreviations

The following abbreviations are used in this manuscript:

PS	Pre-Strained
PSN	Pre-Strained and Notched
DN	Double Notched
FEM	Finite Element Method
C.O.V	Coefficient Of Variation
